# Supplementary material for: Psychological counseling in the Italian academic context: Expected needs, activities, and target population in a large sample of students
Source: PLoS One. 2022 Apr 11;17(4):e0266895. doi: 10.1371/journal.pone.0266895 (PMC9000095; doi:10.1371/journal.pone.0266895)
Supplement: S1 Appendix — Tables S1–S4 report test results and three descriptive statistics that aid in the interpretation of the results. (PDF) [file pone.0266895.s001.pdf]

ADDITIONAL TABLES

February 14, 2022

Tables S1-S4 report test results and three descriptive statistics that can help interpretation: (i) the percentage of the total population of students that belong to the specific category (column 2), (ii and iii) the percentage of students in the whole group (group 1 or group 2) and the percentage of students in the cluster that belong to the specific category, for each cluster.

Over-/Under- represented categories are reported in bold/normal characters (significance codes: '***' 0.001, '**' 0.01, and '*' 0.05). Only clusters with more than 5% of the students in the group were subject to subsequent analysis in the main paper. For completeness, the tables below report more results, including clusters with more that 0.5% of the students in the group.

Table S1: **List of over- and under-represented categories in group 1 - Part I** Column “%" reports the percentage of the total population of students for each category. Column “C1" reports for each category the percentage of students in the specific category that belong to cluster “C1", followed by the percentage of students in cluster “C1" that belong to the specific category

(within parentheses and separated by a comma). The same holds for columns “C2", “C3", “C4", “C6".

Category	%	C1	C2	C3	C4	C6
**Expected activities**						
Training on study skills and learning strategies	-	-	-	-	-	-
Didactic tutoring	4.1	-	-	-	(0,0) **	-
Orientation and career services	1.4	-	**(98.3,35.6)*****	(0,0) ***	-	(0,0) *
Empowerment of personal and context adaptation strategies	2.6	-	(0,0) *	(0,0) ***	**(100,73.1)*****	(0,0) **
Group intervention	4.3	-	-	(0,0) ***	-	**(95.4,67,0)*****
Individual counseling	17.9	(0,0) ***	(1.9,8.8) **	**(93.6,19.7)*****	(1.1,5.5) ***	(3.3,9.7) ***
Sharing and managing personal distress	28.1	(0.1,2.9) ***	(2.1,15) ***	**(94.4,31.1)*****	(0.9,6.9) ***	(2.6,11.7) ***
Psychological evaluation	0.4	-	-	(0,0) ***	-	-
Not sure/No answer	32.3	-	-	**(94,36) *****	(0.8,7.6) ***	(0.5,2.4) ***
Insufficient/No knowledge of the service	0.6	**(100,65.7)*****	-	(0,0) ***	-	-
Other	5.3	-	-	**(93.5,5.9) *****	(0.9,1.4) *	(1.9,1.6) **
**Expected target needs**						
Learning disabilities and disabilities	0.3	-	**(100,6.3) *****	(0,0) ***	-	-
Problems with study skills and learning strategies	-	-	-	-	-	-
Didactic tutoring	1.6	-	-	(0,0) ***	-	**(90.8,23.8)*****
Orientation and career services	2.7	-	**(9.2,6.3) ***	-	-	-
Empowerment of personal and context adaptation strategies	9.6	-	-	-	**(6.2,16.6) ****	-
Personal experience of emotional distress	34.9	(0.3,11.42) **	-	**(88.3,36.2)*****	(2.2,21.3) ***	-
Socio-relational distress	1.1	-	-	(0,0) ***	**(97.7,19.7)*****	-
Psychopathological distress	0.9	-	-	(0,0) ***	-	**(91.4,12.9)*****
Not sure/No answer	37.6	-	-	**(91.6,40.5)*****	(1.8,19.3) ***	(2.1,12.9) ***
Insufficient/No knowledge of the service	0.5	**(100,51.4)*****	-	(0,0) ***	-	-
Other	-	-	-	-	-	-

Table S2: **List of over- and under-represented categories in group 1 - Part II**

Category	%	C1	C2	C3	C4	C6
**Expected target population**						
Students w/ disabilities	2.6	-	**(97.1,62.5)******	(0,0) ***	-	(0,0) **
Students w/ learning disabilities	-	-	-	-	-	-
Students experiencing problems w/ their study skills	20.1	-	(1.6,8.1) ***	-	-	-
Students experiencing relationship problems w/ some teachers and/or tech/admin staff	5.7	-	(0.8,1.3) **	-	-	-
Students experiencing interpersonal relationship problems (w/ friends, family, partners)	8.4	-	(1.2,2.5) **	(81.0,8.0) *	**(5.9,13.8)***	**(10.1,13.7)****
Students w/ study-related emotional distress (anxiety, fears, etc)	29.6	-	(1.2,8.8) ***	**(88.4,30.8)******	-	-
Students experiencing temporary psychological distress	24.0	-	(1.2,7.5) ***	**(88.1,24.9) ****	-	-
Students w/ psychopathological problems	-	-	-	-	-	-
**Sex**						
Women	-	-	-	-	-	-
Men	-	-	-	-	-	-
**Age bin**						
17-24	-	-	-	-	-	-
25-29	-	-	-	-	-	-
30-29	-	-	-	-	-	-
over 40	-	-	-	-	-	-
**Type of degree**						
**a ciclo unico**	24.8	-	-	-	(2.6,17.9)*	-
triennale	-	-	-	-	-	-
magistrale	-	-	-	-	-	-
**Student status**						
on-track (will graduate on time)	-	-		-	-	-
off-track	-	-	-	-	-	-
inactive (haven't acquired CFUs, i.e., study credits, or given exams in the last year)	-	-	-	-	-	-
**Disciplinary area**						
Medical	15.2	-	(2.3,8.8) *	-	-	-
Scientific / Technological	20.9	-	-	-	(2.5,14.5)*	**(8.1,27.4) ***
Health	-	-	-	-	-	-
Socio-Humanistic	59.0	-	-	-	**(4.1,67.6)***	(5.5,52.4) *

Table S3: **List of over- and under- represented categories in group 2 - Part I**

**Category**	%	**C2**	**C3**	**C7**	**C9**	**C10**	**C11**	**C12**
**Expected activities**								
Training on study skills & learning strat.	2.6	-	(0,0) ***	-	**(92.9,32.5)***	-	-	(0,0) *
Didactic tutoring	3.3	-	-	**(11.1,33.3) ***	-	-	-	-
Orientation & career services	1.7	-	(0,0) **	-	-	**(100,36)***	-	(0,0) *
Emp. of personal & context adapt. strat.	1.5	-	(0,0) **	-	-	**(100,32)***	-	(0,0) *
Group intervention	2.8	-	(0,0) ***	-	**(100,37.5) ***	-	-	(0,0) ***
Individual counseling	18.7	-	(9.8,4) ***	-	-	-	-	**(78.4,39.6)***
Sharing & managing personal distress	18.9	-	(9.7,4.1) ***	-	(2.9,7.5) *	(1,4) * -		**(82.5,42.1)***
Psychological evaluation	-	-	-	-	-	-	-	-
Not sure/No answer	45.1	**(4.1,76.9) ***	**(88.2,87.9)***	-	(0.8,5) ***	(0.8,8) ***-		(4.9,6) ***
Insuff./No knowledge	-	-	-	-	-	-	-	-
Other	5	-	(18.5,2) **	-	-	-	-	**(62.9,8.4) **
**Expected target needs**								
Learning dis. & dis.	-	-	-	-	-	-	-	-
Problems w/ study skills & learning strat.	2.8	-	(0,0) ***	-	**(93.3,35) ***	-	-	(0,0) ***
Didactic tutoring	1.1	-	(0,0) *	**(100,100)***	-	-	-	-
Orientation & career services	2.8	-	(0,0) ***	-	-	**(80,48) ***	-	(0,0) ***
Emp. of personal and context adaptation strat.	5.1	-	(0,0) ***	-	-	**(14.2,16) ***	-	**(75,10.4) ***
Personal experience of emotional distress	22	-	(0.8,0.4) ***	-	-	-	-	**(87.5,52) ***
Socio-relational distress	1.1	-	(0,0) *	-	-	-	**(100,100)***	-
Psychopathological distress	-	-	-	-	-	-	-	
Not sure/No answer	57.4	**(3.8,92.3)**	**(77.3,98) ***	(0,0) ***	(2.9,22.5) ***	(1,12) ***	(0,0) **	(14.4,22.3)***
Insuff./No knowledge	-	-	-	-	-	-	-	-
Other	7.2	-	(10.3,1.6) ***	-	-	-	-	**(79.5,15.3)***

Table S4: **List of over- and under- represented categories in group 2 - Part II**

Category	%	C2	C3	C7	C9	C10	C11	C12
**Expected target population**								
Students w/ disabilities	2.4	**(100,100)*****	(0,0) ***	-	-	-	-	(0,0) **
Students w/ learning disabilities	5.5	-	**(80,9.7) *****	-	-	-	-	(6.7,1.0) ***
Students exp. problems w/ their study skills	25.1	(0,0) *	**(65.7,36.4)*****	**(2.9,66.7) ***	-	-	-	(18.3,12.4)***
Students exp. relationship problems w/ some teachers &/or tech/admin staff	6.8	-	**(62.2,9.3) ***	-	-	-	-	-
Students exp. interpersonal relationship problems	8.4	-	(19.6,3.6) ***	-	-	-	-	**(60.9,13.9)*****
Students w/ study-related emotional distress	22.9	(0,0) *	(24.8,12.6)***	-	-	-	-	**(56.8,35.2)*****
Students experiencing temporary psychological distress	20.7	(0,0) *	(33.6,15.4) **	-	-	-	-	**(50.4,28.2) ****
Students w/ psychopathological problems	8.1	-	**(72.7,13.0)*****	-	(0,0) *	-	-	(22.7,5.0) *
**Sex**								
Women	63.7	-	(40.9,57.5) **	-	-	-	-	**(42.1,72.3) ****
Men	36.3	-	**(53.0,42.5) ****	-	-	-	-	(28.3,27.7) **
**Age bin**								
17-24	67.2	-	**(50.3,74.5)*****	-	-	-	-	(32.5,58.9) **
25-29	20.6	-	(32.1,14.6) **	-	-	-	-	**49.1,27.2) ****
30-29	-	-	-	-	-	-	-	-
over 40	2.8	**(20,23.1) ****	-	-	-	-	-	-
**Type of degree**								
a ciclo unico	19.1	-	(35.6,15.0) *	-	-	-	-	**48.1,24.8) ***
triennale	59.8	-	**(50.6,66.8) ****	-	-	-	**(1.8,100)***	(31.3,50.5)***
magistrale	-	-	-	-	-	-	-	-
**Student status**								
on-track	68.3	-	**(49.5,74.5) ****	-	(5.7,52.5)*	**(5.9,88)***	-	(32.5,59.9) **
off-track	28.4	-	(36.8,23.1) *	-	-	-	-	**49,37.6) *****
inactive	3.3	-	-	**(11.1,33.3)***	**(22.2,10)***	-	-	-
**Disciplinary area**								
Medical	-	-	-	-	-	-	-	-
Sci/Tech	-	-	-	-	-	-	-	-
Health	6.8	-	**(64.9,9.7) ***	-	-	-	-	-
Socio-Humanistic	-	-	-	-	-	-	-	-
