# Supplementary material for: Psychological counseling in the Italian academic context: Expected needs, activities, and target population in a large sample of students
Source: PLoS One. 2022 Apr 11;17(4):e0266895. doi: 10.1371/journal.pone.0266895 (PMC9000095; doi:10.1371/journal.pone.0266895)
Supplement: Attachment — Submitted filename: Review report.docx [file pone.0266895.s002.docx]

- **Title**: Capitalize the major variables
- **Line 5-10:** is one sentence – too long, should be broken into 2
- Line 10-15: too long
- Line 15- 19: too long
- Line 50: Since APA is a reference (should be treated as a reference)
- There should be uniformity in referencing – 66 & 67.
- Line 147-153: usage of person centered approach in this study does not come out clearly.
- Line 161: do the authors imply that there is research with a similar approach in the international context? –it is not clear
- Data collection procedure is clearly stated

Hierarchical clustering and inertia gain

Line 257 – 284: not clear, should be explained in the context of the study. Data collection procedure is clear but not data analysis; should be clearly and simply explained to enable even a non biostastician to understand analysis procedures applied.

Main analysis

Line 331-350: presents findings for question 1 – 4; summary at the end of this section will help in understanding the section better.

Line 362-365: A hypothesis is reported to have been tested but earlier in the methodology there was no hypothesis stated. It is important to state it earlier and test it in this section. Again no results are presented of the hypothesis tested and what inferences made from such findings.

Section 2.2: Main Analysis Interpreted

Should be interpreted to allow wider readability. There is need to explain the findings and give its implications. The authors have concentrated on the process without implication which leaves the findings confusing. The authors need to ask themselves who the expected readers are – who are they targeting and whether they will understand the findings; what do they hope the findings will produce?

Pg. 18 line 413-424: These results should be presented and interpreted to be able to make sense.

Page 20: Generally discussion section was well done.

Line 466: the term discipline should be deleted.

Line 481-486: Do the authors suggest that the study influenced the re-organization or did the re-organization influence the study?

Line 648: Hanging sub-title – should it be there if there are no acknowledgements? Study has no summary and conclusions that support data.

References

Well done, only needs uniformity. Choose one style of referencing and use it – do not mix. Authors should use the supporting materials e.g. dendrogram, percentage of explained inertia to interpret the results and give implication.

This article should also fully adhere to the PLO’s guidelines.
