# Supplementary material for: Psychological counseling in the Italian academic context: Expected needs, activities, and target population in a large sample of students
Source: PLoS One. 2022 Apr 11;17(4):e0266895. doi: 10.1371/journal.pone.0266895 (PMC9000095; doi:10.1371/journal.pone.0266895)
Supplement: Attachment — Submitted filename: Response to Reviewers.docx [file pone.0266895.s003.docx]

February 12, 2022

Ref: Manuscript ID **PONE-D-21-25129**

Manuscript Title: “Psychological counseling in the Italian academic context: expected needs,

activities and target population in a large sample of students”.

Dear Mohammed Saqr, Ph.D

Academic Editor

PLOS ONE

thank you very much for offering us the opportunity to submit a revision of the above-titled manuscript. We would like to express our sincerest gratitude to the Reviewers and yourself for your constructive and thoughtful feedback and suggestions.

We have extensively modified the manuscript following the reviewers’ comments and professionally (by <https://www.proof-reading-service.com/en/>) edited the English (American spelling) for clarity. All changes except minor English revisions (to guarantee text fluency) have been highlighted using tracked changes in the “Revised Manuscript with Track Changes” file. We explained how each editorial feedback was addressed in this document.

**Description of, and responses to, Journal Requirements**

**(Query 1).** Please ensure that your manuscript meets PLOS ONE’s style requirements, including those for file naming.

***(Response 1).*** Thanks for this suggestion. We checked the requirements and applied them throughout the manuscript.

***(Query 2).*** You indicated that ethical approval was not necessary for your study. Could you please provide further details on why your study is exempt from the need for approval and confirmation from your institutional review board or research ethics committee (e.g., in the form of a letter or email correspondence) that ethics review was not necessary for this study? Please include a copy of the correspondence as an “Other” file.

***(Response 2).*** Thank you for this note. Although ethical approval was not necessary, following your request, we requested a post-hoc evaluation of the study to the Department’s Ethics Committee which we now attach to the submission form. We mentioned the approval in the paper in section “Data collection procedure”.

***(Query 3).*** Please change “female” or “male” to “woman” or “man” as appropriate, when used as a noun (see for instance <https://apastyle.apa.org/style-grammar-guidelines/bias-free-language/gender>).

***(Response 3).*** We did it throughout the manuscript.

***(Query 4).*** We note that you have stated that you will provide repository information for your data at acceptance. Should your manuscript be accepted for publication, we will hold it until you provide the relevant accession numbers or DOIs necessary to access your data. If you wish to make changes to your Data Availability statement, please describe these changes in your cover letter and we will update your Data Availability statement to reflect the information you provide.

***(Response 4).*** Thanks for this remark. The private link to the repository is: <https://figshare.com/s/e24f094a4c4062425060>. If the paper is accepted we will make this repository public, using the following (reserved) doi <https://doi.org/10.6084/m9.figshare.19092101>. We already included the public (and now not fully available) link in section “Data collection procedure”.

“Data collected for the current study are publicly available [30]

[…] https://figshare.com/s/e24f094a4c4062425060).”

***(Query 5).*** Please include captions for your Supporting Information files at the end of your manuscript, and update any in-text citations to match accordingly. Please see our Supporting Information guidelines for more information: <http://journals.plos.org/plosone/s/supporting-information>.

***(Response 5).*** We implemented the suggested modifications both in the paper and in the Supporting Information files.

**Comments of, and responses to, Reviewer 1**

***(Query 1).*** The authors report the results from the survey conducted in a college in Italy in relation to the use of the university psychological counseling. I was initially excited to learn about ways to improve mental health on college campus, but the results reported in the study are unsurprising and appear to be not so helpful in solving actual problems involving mental health issues in colleges. To summarize the findings, I’ll just quote from the paper; “a picture emerged of what students perceived as main targets of a counseling service: students characterized by study-related emotional distress (with symptoms of anxiety, fear, etc.), by problems with study skills, and/or by temporary psychological distress.” None of these is surprising. And the implications from this finding, “Consistently, the needs targeted by the psychological counseling service are expected to be mainly related to overcoming personal emotional distress, strengthening personal adaptation strategies and the university context and supporting study skills and learning strategies.” are also unsurprising. The other finding from the study is, “the service is expected to be active especially in providing a safe context where to share personal distress and find support to manage it, individual clinical interviews and educational tutoring.” Again, that’s something to be expected. It’s unclear how useful these findings would be for other colleges in improving their own psychological counseling**.**

***(Response 1).***

We understand the Reviewer’s concern. The study intended primarily to adopt a bottom-up approach to the study of students’ perceptions and expectations on the service, given the available large data sample. Furthermore, the study intended to combine the bottom-up approach with a top-down approach to explore whether the two different approaches match and reach the same conclusions.

Our study seems to provide a partial confirmation, as the top-down professional and scientific guidelines on what psychological counseling should be in academic contexts seem to meet students’ perceptions and expectations that emerged from the bottom-up approach, independently from age, sex, academic courses and profiles.

On the other side, it is also true that, on many topics, about half of the respondents did not have a clear idea or had a common and undifferentiated opinion (see for instance analysis results for research question 2, on subsample 1), which seems to explain the existing gap between the actual need for mental health counseling among young adults and the effective use of the counseling service, as clearly highlighted in the introduction.

While very explorative, to the best of our knowledge this is one of the first study exploring students’ perceptions and expectations on psychological counseling services in a very large sample (almost corresponding to the entire population of a large Italian university). The study gives credit to the scientific and professional guidelines on the services; furthermore, it reveals the need of a massive communication campaign, that might promote more awareness of the service and might encourage young students to seek help.

We made this point clearer in the manuscript. Please see the following sentence at the end of the discussion:

“Notwithstanding these limitations, the study makes a new contribution by exploring UPC through a bottom-up approach, that is through students' perceptions and expectations of the service and testing whether this approach matches the top-down one defined by scientific and professional associations' guidelines. Our results seem to provide a partial confirmation, as the top-down professional and scientific guidelines on psychological counseling in academic contexts overlap with students' perceptions and expectations in a way largely independent of their age, sex, academic courses, and profile. On the other hand, it is also true that regarding many themes, about half of the respondents did not have a clear idea or revealed a common and undifferentiated idea about UPC, which could in part explain the existing gap between young adults' need for mental health counseling and the effective use of the counseling service.”

**Comments of, and responses to, Reviewer 2**

***(Query 1).*** Title: Capitalize the major variables

***(Response 1).*** Thanks for suggestion. Although this is a possibility we would also prefer, PLOS ONE guidelines clearly state that capital letters should not be used within the title.

***(Query 2).*** **Line 5-10:** is one sentence – too long, should be broken into 2

***(Response 2).*** Thank you for this note. We changed the sentence as suggested.

***(Query 3).*** Line 10-15: too long

***(Response 3).*** Thank you for this note. We shortened the sentence as suggested.

***(Query 4).*** Line 15- 19: too long

***(Response 4).*** Thank you for this note. We shortened the sentence as suggested.

***(Query 5).*** Line 50: Since APA is a reference (should be treated as a reference)

***(Response 5).*** Thank for noting this. We inserted the reference in the manuscript, as follows:

“American Psychological Association. Counseling psychology. 2008 [cited 17 January 2022]. Available from: <https://www.apa.org/ed/graduate/specialize/counseling>“

***(Query 6).*** There should be uniformity in referencing – 66 & 67.

***(Response 6).*** Thanks for pointing this out. We modified the manuscript following your suggestion.

***(Query 7).*** Line 147-153: usage of person-centered approach in this study does not come out clearly.

***(Response 7).*** Thanks for this remark. We tried to better explain how we applied the person-centered approach in our study. In the same section, we wrote:

“In this line, to answer research question 2, we sought to group students with similar response configurations to questions related to the three expectancy variables (expected target students, expected target needs, and expected target activities). These variables, in fact, can be thought of as “nonlinear" in their relations to each other and to the outcome of actually using or not using the UPC. For example, in one of the authors' practices of managing UPC, expectations about the type of student who accesses the services are not necessarily proportional (and, therefore, linear) to expectations about the type of needs UPC should address or the type of activities that should be provided.”

***(Query 8).*** Line 161: do the authors imply that there is research with a similar approach in the international context? –it is not clear

***(Response 8).*** Yes, it does. There are a few works that adopt a similar approach in the international context. We thank the reviewer for noting that this sentence was not clear. We tried to better explain our concept through the following revised text:

“To our knowledge, there are only a few studies with a similar approach in the international context (see, for example, [18]) and there are none in the Italian one.”

***(Query 9).*** Data collection procedure is clearly stated

***(Response 9).*** Thank you for this positive feedback.

***(Query 10).* (**Hierarchical clustering and inertia gain). Line 257 – 284: not clear, should be explained in the context of the study. Data collection procedure is clear but not data analysis; should be clearly and simply explained to enable even a non biostastician to understand analysis procedures applied.

***(Response 10).*** We extensively modified the description of the methods, trying to explain concepts more clearly and trying to better explain how the methods were applied to the available data and in the given context.

***(Query 11).*** (Main analysis). Line 331-350: presents findings for question 1 – 4; summary at the end of this section will help in understanding the section better.

***(Response 11).*** We agree with the reviewer. Thus, we added the suggested section. You can read in the revised manuscript:

“In summary, most students identified students with emotional distress, mostly related to studying, as the typical target of UPC. Also, most students were unaware of both the needs that UPC addresses and the activities that UPC offers. Despite this general result, a minority of students seemed to recognize UPC goals and that is thus represents a valuable resource. A synergistic collaboration can be imagined between this minority of students and appropriate information campaigns and interventions (see Discussion).”

***(Query 12).*** (Main analysis). Line 362-365: A hypothesis is reported to have been tested but earlier in the methodology there was no hypothesis stated. It is important to state it earlier and test it in this section. Again no results are presented of the hypothesis tested and what inferences made from such findings.

***(Response 12).*** Thank you for your note. The hypothesis tested is “that the proportion of the population belonging to a category within a cluster is equal to the proportion of the population belonging to the chosen category in the whole population.” In other words, the test will answer the question: “is a category over or underrepresented in the given cluster?”, or equivalently, “were students in a given cluster characterized differently than the average student?” We have moved the indicated sentences to the “Data analysis procedure” section, keeping a small catch phrase in the initial position. Regarding the results on this point, it seems to us that we made them explicit in section “Group 1”, where we report:

“The variables that characterize group 1 are “Expected target needs" (p-value < 0.001), “Expected activities" (p-value < 0.001), “Expected target students" (p-value < 0.001), “Disciplinary area" (p-value < 0.05) as obtained with a χ^2^ test. The set of variables that characterize the clusters, and are therefore significant in defining the different profiles that emerge from the group, are listed in Tables S1 and S2 of the supplementary materials.”

***(Query 13).*** (Main Analysis Interpreted). Should be interpreted to allow wider readability. There is need to explain the findings and give its implications. The authors have concentrated on the process without implication which leaves the findings confusing. The authors need to ask themselves who the expected readers are – who are they targeting and whether they will understand the findings; what do they hope the findings will produce?

***(Response 13).*** Thank you for this note. We usually consider the results section to be strongly descriptive, and present interpretations in the discussion section. In any case, we have made an effort to add a summary and a very brief interpretation in the analysis section. See Response 14.

As to the issue of who the expected readers are and what is the take home message, the study attempts to contribute to the understanding of the existing gap between the need for counseling for mental health problems among young adults and the effective use of the counseling service, as highlighted in then introduction. Different reasons might explain such gap, such as the services not matching adequately students’ needs and/or college students not having a clear representation of what the service is precisely. Given that the study has both a basic and a translational research purpose, we believe our target readers are quite wide. We tried to make this point clearer in the discussion section. Please see the sentence reported below at the end of the discussion:

“Notwithstanding these limitations, the study makes a new contribution by exploring UPC through a bottom-up approach, that is through students' perceptions and expectations of the service and testing whether this approach matches the top-down one defined by scientific and professional associations' guidelines. Our results seem to provide a partial confirmation, as the top-down professional and scientific guidelines on psychological counseling in academic contexts overlap with students' perceptions and expectations in a way largely independent of their age, sex, academic courses, and profile. On the other hand, it is also true that regarding many themes, about half of the respondents did not have a clear idea or revealed a common and undifferentiated idea about UPC, which could in part explain the existing gap between young adults' need for mental health counseling and the effective use of the counseling service.”

***(Query 14).*** (Main Analysis Interpreted). Pg. 18 line 413-424: These results should be presented and interpreted to be able to make sense.

***(Response 14).*** We agree with the Reviewer that a brief summary to focus on the main results of aim of research question 2 is necessary, similarly to what done for research question 1. Therefore, we have added a brief summary at the end of the section as follows:

“In summary, we used clustering analysis and the obtained dendrograms reported in the Supplementary Materials to identify further subgroups of Group 1 and Group 2. Then, after we identified the subgroups, we proceeded with a statistical analysis to characterize them. We observed that students in Group 1 and students in Group 2 had two different mental representations of the service. On one side, students who expressed their intention to use the counseling service but had not yet done so (Group 1), had a less differentiated, narrower and more common perception of the service, mainly adjusted to an emotional and a psychopathological focus, without a clear idea of the needs the service should target. On the other side, students who had accessed the service in the past (Group 2), had a more articulated representation of the service, and expected a service that adapts with flexibility to a variety of target populations and needs including clinical, learning, and socioemotional areas.”

***(Query 15).*** (Main Analysis Interpreted). Page 20: Generally, discussion section was well done.

***(Response 15).*** Thanks for this positive feedback.

***(Query 16).*** (Main Analysis Interpreted). Line 466: the term discipline should be deleted.

***(Response 16).*** Thank you for the note. We replace the term in the text.

***(Query 17).*** (Main Analysis Interpreted). Line 481-486: Do the authors suggest that the study influenced the re-organization or did the re-organization influence the study?

***(Response 17).*** Thank you for requesting this clarification. What we meant is that the results of this study influenced the reorganization of the psychological counselling service. Accordingly, in the revised manuscript, you can read:

“Accordingly, it must be underlined that this investigation took place at, and influenced, the beginning of a massive reorganization of the psychological service in the university context where the research took place.”

***(Query 18).*** (Main Analysis Interpreted). Line 648: Hanging sub-title – should it be there if there are no acknowledgements? Study has no summary and conclusions that support data.

***(Response 18).*** Thanks for these suggestions. We deleted the Acknowledgements section. Also, we added a Conclusions section:

“In recent years, UPC has received much attention as a possible means of promoting mental health, academic success, and, in the long term and on a large scale, the growth of society. Our study has tried to contribute to the existing literature on the implementation and dissemination of the practice of psychological counseling in the university context, with a bottom-up approach complementary to the usual top-down one that follows the guidelines of scientific-professional associations for counseling. The results reveal that a large majority of students do not have a clear idea both about the needs that UPC should address and the activities it should offer. However, about half of them were able to adequately identify university students as the target population of UPC. These latter students can be considered an important resource for active involvement in awareness and prevention campaigns aimed at their peers in order to increase the use of UPC and consequently reduce the impact of mental health problems among university students. Considering two specific subsamples of the study participants, namely (i) those who only expressed their intention to use the counseling service and (ii) those who had already used it, different student profiles are found in the two groups. For the first group, a profile with an "emotional" focus and one with a "psychopathological" focus were identified; in the second group, a profile with a "clinical" focus, one with a "socioemotional" focus and one with a "learning" focus were identified. This picture of the results suggests that in the former there is a fairly \superficial" and \common" representation of UPC, while in the latter this representation is more \articulated" and \flexible." This second series of results confirms the need for intense communication campaigns, even among students who intend to use counseling but have not yet done so. Such campaigns should better clarify the specific characteristics of the service and could use IT channels, which are widely used among students. Overall, the findings of this study suggest the importance of adopting student-centered strategies both to reach a wider audience and to identify, and therefore respond, to students' specific expectations and needs. Furthermore, these findings align with top-down approaches to identify the variables that are important for a successful UPC service. Nonetheless, further studies adopting both approaches are needed and expected in this research area.”

***(Query 19).*** Authors should use the supporting materials e.g. dendrogram, percentage of explained inertia to interpret the results and give implication.

***(Response 19).*** MCA, clustering, dendrograms and percentage of explained inertia are used to identify subgroups of Groups 1 and 2. Afterwards, the obtained subgroups are characterized using a statistical test described in section “Data analysis procedure”, that identifies categories that are over or under-represented (and therefore “characterize” the subgroups). As a result, clustering and MCA analysis are instrumental for subsequent analysis and their efficacy is proven by the interpretability of the resulting subgroups (e.g. a subgroup with clinical focus, a subgroup with psychopathological focus, etc.).

“In summary, we used clustering analysis and the obtained dendrograms reported in the Supplementary Materials to identify further subgroups of Group 1 and Group 2. Then, after we identified the subgroups, we proceeded with a statistical analysis to characterize them.”

***(Query 20).*** Well done, only needs uniformity. Choose one style of referencing and use it – do not mix.

***(Response 20).*** Thanks, we checked and used the PLO’s reference style.

***(Query 21).*** This article should also fully adhere to the PLO’s guidelines.

***(Response 21).*** Thanks for the suggestion. We fully checked the manuscript following PLO’s guidelines, as also suggested by the Editor.
